# Food Safety: A Tea-Time Mystery

**Published:** 2005-08

**Authors:** Michael Szpir

When a 52-year-old Missouri woman approached physicians in 1998 complaining of stiffness and pain in her spine, the symptoms were at first attributed to “disc disease.” But a series of laboratory tests showed that the woman had abnormally thick, dense bones and strikingly high levels of fluoride in her urine—hallmarks of skeletal fluorosis, a disease that has been diagnosed only a handful of times in the United States.

The only way to develop skeletal fluorosis is to ingest or inhale too much fluoride. The woman’s drinking water had only about 2.8 parts per million (ppm) fluoride, well below the Environmental Protection Agency (EPA) limit of 4.0 ppm. Other sources of fluoride were also eliminated: She didn’t swallow her toothpaste, she didn’t work with pesticides, and she didn’t live near a mine. So where was she getting all the fluoride?

Then the woman revealed she had drunk up to two gallons of extra-strength instant tea every day of her adult life. Physician Michael Whyte of Washington University School of Medicine and his colleagues decided to measure the fluoride levels in her tea preparation.

They found that, counting the fluoride in her water, the woman was ingesting 37–74 milligrams of fluoride per day. EPA studies suggest that severe skeletal fluorosis could occur over the course of 20 years from a continuous exposure of 20 milligrams of fluoride per day.

Whyte and colleagues then tested 10 instant teas available in grocery stores. They found average fluoride concentrations of 1.0–6.5 ppm in regular-strength tea made with fluoride-free water, with several brands exceeding the Food and Drug Administration limit of 1.4–2.4 ppm for bottled beverages. Their study appears in the January 2005 issue of *The American Journal of Medicine*.

Whyte believes that individuals who drink large volumes of instant tea for a prolonged period may be putting themselves at risk for skeletal fluorosis. But Joe Simrany, president of The Tea Association of the USA, believes that the Missouri incident was highly unusual. “It had less to do with tea than it had to do with excessive behavior,” he says.

So should the average tea drinker be concerned? “It may be that certain brands ought to cut down the amount of fluoride in their tea or add a warning label to their product,” says Michael Kleerekoper, director of research for bone and mineral metabolism at Wayne State University, “but it would be a real mistake to throw out the baby with the bathwater.” He adds, “I drink tea—it’s wonderful on a hot summer’s afternoon.”

Whyte, who also hasn’t stopped drinking tea, says, “Our research is a call for better understanding of fluoride levels in various teas.” He is now investigating the fluoride levels of bottled tea preparations.

Meanwhile, the woman in Missouri has stopped drinking tea, and her pains have abated. She has since switched to lemonade.

## Figures and Tables

**Figure:**
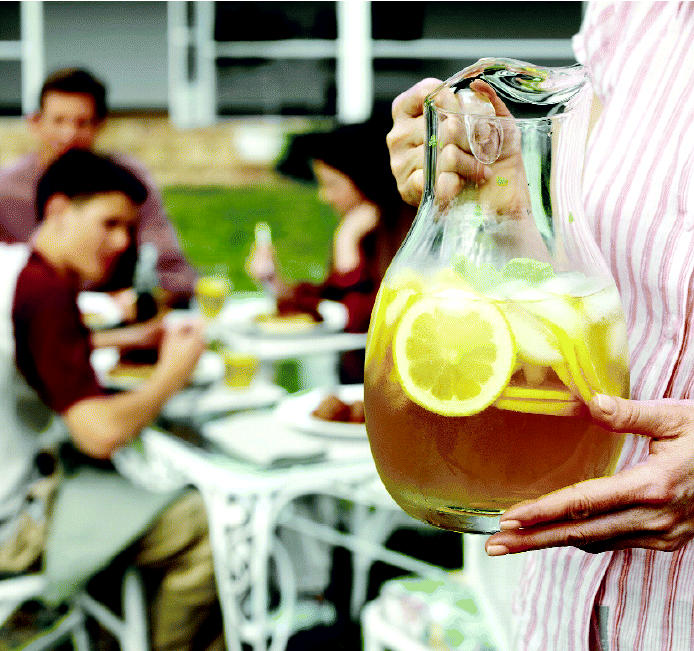
Tea total. Some instant teas may exceed safe levels of fluoride, suggesting a little refreshment goes a long way.

